# Supplementary material for: Non-small cell lung cancer microbiota characterization: Prevalence of enteric and potentially pathogenic bacteria in cancer tissues
Source: PLoS One. 2021 Apr 23;16(4):e0249832. doi: 10.1371/journal.pone.0249832 (PMC8064568; doi:10.1371/journal.pone.0249832)
Supplement: S2 Fig — Each section of the bars represents one OTU. Each bacterial genus is identified with a different color. The type of tissue, cancerous or healthy, is specified by a full or dotted outline, respectively. (DOCX) [file pone.0249832.s002.docx]

**S2 Fig. Relative abundance and taxonomic identification of OTUs shared between the two tissues samples from a same patient not correlated by Pearson’s test (p-value>0.05).** Each section of the bars represents one OTU. Each bacterial genus is identified with a different color. The type of tissue, cancerous or healthy, is specified by a full or dotted outline, respectively.
