# Supplementary material for: Non-small cell lung cancer microbiota characterization: Prevalence of enteric and potentially pathogenic bacteria in cancer tissues
Source: PLoS One. 2021 Apr 23;16(4):e0249832. doi: 10.1371/journal.pone.0249832 (PMC8064568; doi:10.1371/journal.pone.0249832)
Supplement: S3 Fig — The numbers in parentheses represent the sum of the average relative abundance of the OTUs in the samples. The non-core score is the number of OTUs with relative abundances higher than 0.001% that are not present in at least 30% of the sample one of the categories. (DOCX) [file pone.0249832.s003.docx]

Cancerous

AC

Cancerous

SqCC

**S3 Fig. Number of OTUs shared by 30% of all cancerous tissues for each type of cancer with relative abundances higher than 0.001%.** The numbers in parentheses represent the sum of the average relative abundance of the OTUs in the samples. The non-core score is the number of OTUs with relative abundances higher than 0.001% that are not present in at least 30% of the sample one of the categories.
