# Supplementary material for: Non-small cell lung cancer microbiota characterization: Prevalence of enteric and potentially pathogenic bacteria in cancer tissues
Source: PLoS One. 2021 Apr 23;16(4):e0249832. doi: 10.1371/journal.pone.0249832 (PMC8064568; doi:10.1371/journal.pone.0249832)
Supplement: S5 Fig — A. Shannon’s alpha diversity index. B. Pielou’s evenness. Doubled-sided t-tests paired by patients were performed. The boxes display the data range, quartiles and median. The mean is displayed as white lozenges. (DOCX) [file pone.0249832.s005.docx]

**Alpha Diversity Analyses**

The mean of Shannon’s diversity index was significantly higher (p=0.041) in cancerous samples of patients with adenocarcinoma, but equivalent in patient with squamous cell carcinoma (figure 3A). The increased evenness of OTUs distribution was identified as the driving force of this trend since Shannon index incorporate calculation of the number of OTUs and their distribution (figure 3B). The Pielou’s evenness were computed using the R package “microbiome” ^45^. The number of rarefied OTUs were similar between tissues and non-rarefied numbers are available in table 2 or the article. Tissues coming from patients with squamous cell carcinoma tend to be more diverse than those of adenocarcinoma, but the high variability between samples and the reduced statistical power of non-paired tests lead to no significant differences (p>0.05) (figures 5-6).

**S5 Fig. Alpha diversity of tissue samples by type of cancer.** **A.** Shannon’s alpha diversity index. **B.** Pielou’s evenness. Doubled-sided t-tests paired by patients were performed. The boxes display the data range, quartiles and median. The mean is displayed as white lozenges.
