# Supplementary material for: Non-small cell lung cancer microbiota characterization: Prevalence of enteric and potentially pathogenic bacteria in cancer tissues
Source: PLoS One. 2021 Apr 23;16(4):e0249832. doi: 10.1371/journal.pone.0249832 (PMC8064568; doi:10.1371/journal.pone.0249832)
Supplement: S6 Fig — Doubled-sided non-paired t-tests were performed. The boxes display the data range, quartiles and median. The mean is displayed as white lozenges. (DOCX) [file pone.0249832.s006.docx]

**S6 Fig. Comparison of Shannon’s diversity index of tissue samples by type of cancer**. Doubled-sided non-paired t-tests were performed. The boxes display the data range, quartiles and median. The mean is displayed as white lozenges.
