# Supplementary material for: Non-small cell lung cancer microbiota characterization: Prevalence of enteric and potentially pathogenic bacteria in cancer tissues
Source: PLoS One. 2021 Apr 23;16(4):e0249832. doi: 10.1371/journal.pone.0249832 (PMC8064568; doi:10.1371/journal.pone.0249832)
Supplement: S1 Table — (DOCX) [file pone.0249832.s007.docx]

**S1 Table. Accession number of the raw sequencing data in the project PRJNA680529 of the NCBI Sequence Read Archive.**

| NCBI Accession Number | Sample Name | NCBI Accession Number | Sample Name |
| --- | --- | --- | --- |
| SAMN16881876 | Patient1_Control | SAMN16881921 | Patient16_Control |
| SAMN16881877 | Patient1_Healthy | SAMN16881922 | Patient16_Healthy |
| SAMN16881878 | Patient1_Cancerous | SAMN16881923 | Patient16_Cancerous |
| SAMN16881879 | Patient2_Control | SAMN16881924 | Patient17_Control |
| SAMN16881880 | Patient2_Healthy | SAMN16881925 | Patient17_Healthy |
| SAMN16881881 | Patient2_Cancerous | SAMN16881926 | Patient17_Cancerous |
| SAMN16881882 | Patient3_Control | SAMN16881927 | Patient18_Control |
| SAMN16881883 | Patient3_Healthy | SAMN16881928 | Patient18_Healthy |
| SAMN16881884 | Patient3_Cancerous | SAMN16881929 | Patient18_Cancerous |
| SAMN16881885 | Patient4_Control | SAMN16881930 | Patient19_Control |
| SAMN16881886 | Patient4_Healthy | SAMN16881931 | Partient19_Healthy |
| SAMN16881887 | Patient4_Cancerous | SAMN16881932 | Patient19_Cancerous |
| SAMN16881888 | Patient5_Control | SAMN16881933 | Patient20_Control |
| SAMN16881889 | Patient5_Healthy | SAMN16881934 | Patient20_Healthy |
| SAMN16881890 | Patient5_Cancerous | SAMN16881935 | Patient20_Cancerous |
| SAMN16881891 | Patient6_Control | SAMN16881936 | Patient21_Control |
| SAMN16881892 | Patient6_Healthy | SAMN16881937 | Patient21_Healthy |
| SAMN16881893 | Patient6_Cancerous | SAMN16881938 | Patient21_Cancerous |
| SAMN16881894 | Patient7_Control | SAMN16881939 | Patient22_Control |
| SAMN16881895 | Patient7_Healthy | SAMN16881940 | Patient22_Healhty |
| SAMN16881896 | Patient7_Cancerous | SAMN16881941 | Patient22_Cancerous |
| SAMN16881897 | Patient8_Control | SAMN16881942 | Patient23_Control |
| SAMN16881898 | Patient8_Healthy | SAMN16881943 | Patient23_Healthy |
| SAMN16881899 | Patient8_Cancerous | SAMN16881944 | Patient23_Cancerous |
| SAMN16881900 | Patient9_Control | SAMN16881945 | Patient24_Control |
| SAMN16881901 | Patient9_Healthy | SAMN16881946 | Patient24_Healthy |
| SAMN16881902 | Patient9_Cancerous | SAMN16881947 | Patient24_Cancerous |
| SAMN16881903 | Patient10_Control | SAMN16881948 | Patient25_Control |
| SAMN16881904 | Patient10_Healthy | SAMN16881949 | Patient25_Healthy |
| SAMN16881905 | Patient10_Cancerous | SAMN16881950 | Patient25_Cancerous |
| SAMN16881906 | Patient11_Control | SAMN16881951 | Patient26_Control |
| SAMN16881907 | Patient11_Healthy | SAMN16881952 | Patient26_Healthy |
| SAMN16881908 | Patient11_Cancerous | SAMN16881953 | Patient26_Cancerous |
| SAMN16881909 | Patient12_Control | SAMN16881954 | Patient27_Control |
| SAMN16881910 | Patient12_Healthy | SAMN16881955 | Patient27_Healthy |
| SAMN16881911 | Patient12_Cancerous | SAMN16881956 | Patient27_Cancerous |
| SAMN16881912 | Patient13_Control | SAMN16881957 | Patient28_Control |
| SAMN16881913 | Patient13_Healthy | SAMN16881958 | Patient28_Healthy |
| SAMN16881914 | Patient13_Cancerous | SAMN16881959 | Patient28_Cancerous |
| SAMN16881915 | Patient14_Control | SAMN16881960 | Patient29_Control |
| SAMN16881916 | Patient14_Healthy | SAMN16881961 | Patient29_Healthy |
| SAMN16881917 | Patient14_Cancerous | SAMN16881962 | Patient29_Cancerous |
| SAMN16881918 | Patient15_Control |  | |
| SAMN16881919 | Patient15_Healthy |  |  |
| SAMN16881920 | Patient15_Cancerous |  |  |
